# Roles of mTOR in Diabetic Kidney Disease

**DOI:** 10.3390/antiox10020321

**Published:** 2021-02-22

**Authors:** Mako Yasuda-Yamahara, Shinji Kume, Hiroshi Maegawa

**Affiliations:** Department of Medicine, Shiga University of Medical Science, Tsukinowa-cho, Seta, Otsu, Shiga 520-2192, Japan; maegawa@belle.shiga-med.ac.jp

**Keywords:** mTORC1, diabetic kidney disease, oxidative stress, podocyte, proximal tubular cell

## Abstract

Diabetic kidney disease (DKD) is the leading cause of end-stage renal disease and the number of patients affected is increasing worldwide. Thus, there is a need to establish a new treatment for DKD to improve the renal prognosis of diabetic patients. Recently, it has shown that intracellular metabolic abnormalities are involved in the pathogenesis of DKD. In particular, the activity of mechanistic target of rapamycin complex 1 (mTORC1), a nutrient-sensing signaling molecule, is hyperactivated in various organs of diabetic patients, which suggests the involvement of excessive mTORC1 activation in the pathogenesis of diabetes. In DKD, hyperactivated mTORC1 may be involved in the pathogenesis of podocyte damage, which causes proteinuria, and tubular cell injury that decreases renal function. Therefore, elucidating the role of mTORC1 in DKD and developing new therapeutic agents that suppress mTORC1 hyperactivity may shed new light on DKD treatments in the future.

## 1. Introduction

**Figure 1:**
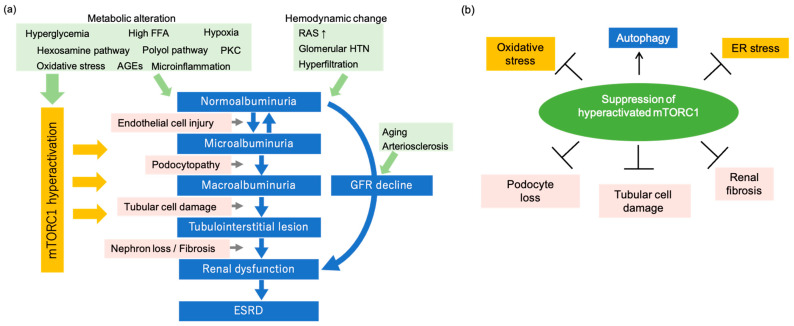
Major pathogenesis and clinical course of diabetic kidney disease (DKD) and effects of mammalian target of rapamycin complex 1 (mTORC1) suppression in DKD. (**a**) In diabetes mellitus patients, metabolic abnormalities such as hyperglycemia, high free fatty acids (FFAs), and hypoxia and hemodynamic abnormalities such as hyperactive renin-angiotensin system (RAS) and hyperfiltration are involved in the progression of DKD. DKD progresses from normoalbuminuria to microalbuminuria and macroalbuminuria, and the tubulointerstitial lesion to renal dysfunction and end-stage renal failure. In recent years, the number of patients with a decreased glomerular filtration rate (GFR) without proteinuria has increased, and aging and atherosclerosis are thought to be involved in the decrease in GFR. Furthermore, mTORC1 activity, which is hyperactivated by metabolic abnormalities, may promote DKD progression. PKC, protein kinase C; AGEs, advanced glycation end-products. (**b**) Amelioration of hyperactivated mTORC1 decreases oxidative stress and ER stress. mTORC1 suppression activates autophagy and prevents podocyte loss, tubular cell damage, and renal fibrosis in DKD.

Diabetic kidney disease (DKD) is one of the most serious complications of diabetes and the leading cause of end-stage renal disease worldwide. The number of patients with DKD is increasing and new therapeutic agents for DKD are urgently needed. The typical clinical course of DKD begins with microalbuminuria, followed by severe proteinuria, which induces tubular damage and eventually leads to a decline in renal function and end-stage renal disease (Figure 1a). In recent years, there has been an increase in the number of diabetic patients who present with decreased renal function without proteinuria [1,2,3]. This condition is thought to involve background factors such as aging and atherosclerosis, and may be different from the typical DKD condition. In clinical practice, the lack of an effective treatment for DKD with refractory proteinuria and the increasing number of patients with new forms of DKD who do not present with proteinuria but with reduced renal function are thought to be contributing factors to the increasing number of patients with DKD that leads to end-stage renal failure. Thus, there is a need to elucidate the pathogenesis of refractory proteinuria, the mechanism of progression from refractory proteinuria to renal function decline, and the pathogenesis of renal function decline without proteinuria.

The pathogenesis of DKD is extremely complex (Figure 1a). The mechanism of renal glomerular cell damage under diabetic conditions has been considered to be hyperglycemia-induced metabolic abnormalities and hemodynamic abnormalities such as hyperfiltration due to systemic and glomerular hypertension associated with a hyperactive renin–angiotensin system (RAS) [4,5,6,7]. Hyperglycemia-induced activation of the polyol pathway, protein kinase C, hexosamine pathway, oxidative stress, and excessive production of advanced glycation end-products act cytotoxically to induce glomerular damage, glomerulosclerosis, and tubular damage. Additionally, the mechanism of DKD is thought to not only involve the abovementioned factors, but also dyslipidemia, hyperinsulinemia, and obesity, as well as multiple factors such as environmental and genetic factors [8,9,10,11]. It has been reported that microinflammation triggered by abnormal fatty acid metabolism, endothelial damage, oxidative stress, decreased autophagy activity, and hypoxia in renal tissue are involved in the development and progression of DKD [12,13,14,15,16,17].

**Figure 2:**
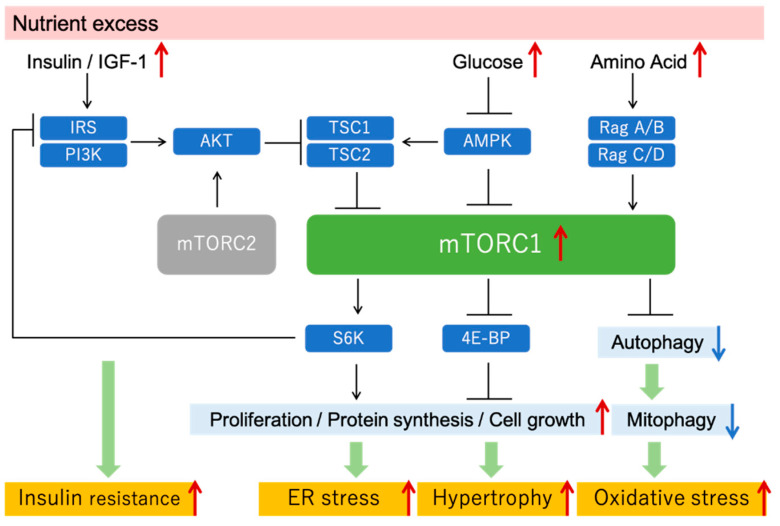
mTOR signaling and mTORC1 hyperactivation under nutrient excess condition. Excessive nutrition, such as that under diabetic conditions, activates mTORC1, which in turn increases protein synthesis and cell proliferation and suppresses autophagy. Inappropriate mTORC1 hyperactivation increases ER stress, tissue hypertrophy, oxidative stress, and insulin resistance. IGF, insulin-like growth factor; IRS, insulin receptor substrate; TSC, tuberous sclerosis complex; 4E-BP, 4E-binding protein.

Accumulating evidence has demonstrated the relationship between alterations in nutrient-sensing signals and DKD progression [8,18,19]. Thus far, we have focused on mechanistic target of rapamycin complex 1 (mTORC1) as a pathway involved in the pathogenesis of DKD. mTOR is a protein kinase conserved in all species from yeasts to mammals, which was discovered in yeast as a binding protein for rapamycin. It forms at least two complexes (mTORC1 and mTORC2) with different binding proteins, each of which performs different functions by phosphorylating different substrates. Growth factors such as insulin, amino acids, and sugars increase mTORC1 activity, and increased mTORC2 activity is mainly regulated by growth factor stimulation. By phosphorylating its substrates, mTORC1 promotes cell differentiation and growth, enhances intracellular anabolism, and inhibits catabolism such as autophagy [20,21]. mTORC2 phosphorylates and activates other kinases mainly as a substrate to promote cytoskeletal reorganization and cell proliferation and inhibit cell death [22]. Interestingly, mTORC1 causes the functional suppression of adaptor proteins that play important roles downstream of insulin receptors and insulin-like growth factor receptors, which is a cause of insulin resistance [23,24,25] (Figure 2).

The increase in the intracellular glucose concentration due to hyperglycemia suppresses AMP kinase activity and activates mTORC1. In addition to hyperglycemia, obesity, and type 2 diabetes, hyperinsulinemia and excess amino acids associated with over-eating are factors required for increased mTORC1 activity, which is observed not only in the kidneys [26,27,28] but also in other organs such as adipose tissue and the liver [29,30,31,32]. Excessive mTORC1 upregulation can lead to the dysregulation of intracellular protein synthesis and the metabolic balance, which increases endoplasmic reticulum stress and intracellular oxidative stress (Figure 2).

In this review, we describe the pathogenesis of DKD by focusing on mTORC1 hyperactivation and discuss the therapeutic potential of mTORC1 suppression for DKD.

## 2. Podocyte Injury in Typical DKD and mTORC1

The main cause of proteinuria is the disruption of the glomerular filtration barrier function. The glomerular filtration barrier consists of three layers: vascular endothelial cells, glomerular basement membrane, and podocytes. In particular, podocytes are highly differentiated terminally differentiated cells that form a foot structure between neighboring cells and play an important role in the glomerular filtration barrier function. Additionally, podocytes have almost no proliferative activity and it is thought that once they are damaged they directly contribute to the failure of the glomerular barrier function. 

The hyperactivation of mTORC1 is observed in DKD podocytes, which causes cytotoxicity. It has been reported that the mTORC1 activity increases in the podocytes of diabetic mice and rats [15,26,27,33]. Podocyte-specific Raptor, an essential protein for mTORC1, heterozygous mice with suppressed mTORC1 activity, show reduced urinary proteins in both STZ-induced type 1 diabetic mice [26] and type 2 diabetic db/db mice [27]. It has also been reported that podocyte-specific mTORC1 hyperactivity model mice with podocyte-specific deletion of tuberous sclerosis complex 1, a protein that suppresses mTORC1, show podocyte damage, proteinuria, and an increased mesangial area similar to the glomerular lesions caused by DKD [27].

Furthermore, mTORC1 negatively regulates autophagy, an intracellular catabolic process. Autophagy degrades damaged organelles and proteins, which is triggered by stress and starvation. Autophagy is necessary to maintain cellular homeostasis. It has been shown that autophagy decreases in the podocytes of DKD patients with massive proteinuria [15]. Furthermore, mice with podocyte-specific autophagy deficiency have severe podocyte damage and massive proteinuria under diabetic conditions [15]. These findings suggest that decreased podocyte autophagy induces podocyte dysfunction and is involved in the progression of DKD. The maintenance of proper autophagy activity in podocytes by mTORC1 correction may be protective for DKD podocytes (Figure 1b).

## 3. Tubular Cell Injury in Typical DKD and mTORC1

In glomerular diseases including DKD, a large amount of urinary proteins leaked from the glomerulus is reabsorbed by the proximal tubules, which overloads the tubular cells and induces inflammation and fibrosis of the tubular interstitium, ultimately leading to declines in renal function (Figure 1a). Interestingly, autophagy is induced in proximal tubular cells by reabsorption of albumin in mice [16]. Additionally, mTORC1 is activated in proximal tubular cells of obese type 2 diabetic mice fed a high-fat diet, which suppresses autophagy induced by urinary proteins [16]. In diabetic proximal tubular cells, the cytoprotective autophagy induced by urinary proteins is suppressed by the activation of mTORC1. As a result, susceptibility to urinary protein-associated cytotoxicity is induced. Rapamycin, an mTORC1 inhibitor, suppresses excessive mTORC1 activity and inhibits tubular cell damage in obese type 2 diabetic mice fed a high fat diet [16]. Moreover, the activation of the mTOR pathway is involved in the increased expression of profibrotic cytokines, such as TGF-β1 and connective tissue growth factor, and subsequent interstitial fibrosis of DKD [34,35]. Furthermore, proximal tubular cell-specific Raptor heterozygous-deficient mice exhibit reduced tubular fibrosis and reduced renal function under diabetic conditions [36]. These results suggest that the abnormal activation of mTORC1 is involved in the pathogenesis of tubular damage in DKD. Therefore, correcting abnormal intracellular nutrient signaling, including the suppression of mTORC1, is expected to be a new therapeutic target for the tubulointerstitial lesion in DKD (Figure 1b).

## 4. Non-Proteinuric DKD and mTORC1

In recent years, many patients with impaired renal function due to diabetes mellitus have shown a decreased GFR without microalbuminuria or proteinuria [1,2]. It has been speculated that arteriosclerotic and tubulointerstitial lesions due to aging and hypertension exist in the background of these patients (Figure 1a). Strict glycemic control and hypertension treatment with renin-angiotensin system inhibitors have prevented the typical progression of DKD from microalbuminuria to proteinuria, tubular damage, and renal function declines [37]. This is thought to be the result of the successful implementation of evidence-based multidisciplinary treatment, as inferred from the number of patients on dialysis due to their diabetes having leveled off. However, a large number of new patients with diabetes mellitus remain on dialysis. There are interesting reports on the clinical picture of DKD patients in recent years. A review of diabetic patients for several decades showed that, although the incidence of albuminuria has decreased, the percentage of patients with reduced eGFR has increased [1]. This observation suggests that, while the progression of renal damage caused by typical DKD, which is triggered by proteinuria due to glomerular lesions, has been decreasing, the effect of the involvement of nephrosclerosis related to aging, hypertension, and arteriosclerosis on GFR decline has been increasing (Figure 1a). Therefore, it is necessary to clarify the pathogenesis of both types of DKD to develop treatment strategies for DKD.

In a study of cultured cells, the mTORC1 activity in cultured tubular cells was suppressed by hypoxic stimuli, but the coadministration of diabetes-like stimuli (hyperglycemia and a high concentration of palmitic acid) under hypoxic conditions increased mTORC1 activity in the cells, which led to cell death. Furthermore, this cell death was inhibited significantly by the addition of rapamycin [38]. These results suggest that mTORC1, which should be suppressed under hypoxic conditions such as atherosclerosis, is excessively upregulated in the diabetic state with hypoxia and this may be involved in cell damage. Additionally, ApoE-deficient high fat diet-overloaded mice exhibit atherosclerosis, tubular damage without proteinuria, and reduced renal function, which have been reported to be useful as a DKD model without proteinuria. In these mice, tubular cells show tubular damage and the hyperactivation of tubular mTORC1 [39]. Furthermore, administration of ketone bodies or SGLT2 inhibitors suppresses mTORC1 hyperactivation in tubules and reduces tubular damage [39]. These findings suggest that excessive mTORC1 activation is involved in the pathogenesis of DKD even in non-proteinuric DKD (Figure 1a). Therefore, mTORC1 suppression may be effective to inhibit the development and progression of non-proteinuric DKD.

## 5. Oxidative Stress and mTORC1

Oxidative stress is a major factor in the pathogenesis of DKD. In the diabetic state, hyperglycemia enhances the production of reactive oxygen species (ROS) in the kidneys. Intracellular metabolic abnormalities, such as hyperglycemia-induced enhancement of the polyol pathway, protein kinase C activation, and the accumulation of intracellular glycated proteins, have been implicated in the increase in ROS in diabetic kidneys [40,41]. High levels of free fatty acids (FFAs) may also stimulate ROS generation under diabetic conditions [42,43]. Moreover, the accumulation of damaged mitochondria in diabetic kidneys contributes to ROS generation [44,45]. Increased oxidative stress indices in blood and urine have been reported in diabetic patients and animals [46,47,48,49], and there have been many reports that suggest an increase in oxidative stress in renal tissues. In STZ rats, studies have reported an increase in the content of 8-hydroxydeoxyguanosine (8-OHdG), an indicator of oxidative stress, in renal tissue, an increase in urinary 8-OHdG excretion, and a significant increase in the 8-OHdG content of mitochondrial DNA in renal tissue [50]. Increased urinary 8-OHdG excretion and increased mitochondrial oxidative damage in renal tissue have also been reported in db/db mice [51]. mTORC1 signaling and oxidative stress are closely related to each other. Insulin resistance induced by excessive mTORC1 activation promotes ROS synthesis. Furthermore, autophagy plays an important role in maintaining mitochondrial functions. Mitochondrial quality control is mediated by mitochondrial autophagy (mitophagy) [52,53,54]. Oxidative stress also induces autophagy to remove damaged mitochondria in an attempt to protect the cell. Thus, autophagy-mediated mitochondrial quality control and the subsequent reduction in ROS may be essential to protect the kidneys of diabetic patients. Therefore, upon autophagy suppression by inappropriate mTORC1 hyperactivation, the decrease in autophagy activity decreases intracellular organelle and mitochondrial functions, which in turn increases oxidative stress (Figure 2). These findings suggest that suppression of excessive mTORC1 activity in diabetic patients may also decrease ROS (Figure 1b).

## 6. Clinical Use and Adverse Effects of mTOR Inhibitors

Several studies have reported the inhibitory effect of rapamycin on the development and progression of DKD in diabetic animal models [34,55,56,57,58,59]. In fact, rapamycin is widely used clinically as an anti-cancer drug and post-transplantation immunosuppressive agent, which may have potential as a DKD treatment. However, it is also true that many side effects have been reported with rapamycin [60]. It has been reported to cause nephrotoxicity, such as increased urinary protein [61], acute tubular necrosis [62], and FSGS [63], as well as metabolic abnormalities such as insulin resistance, glucose intolerance [64,65], and abnormal lipid metabolism [21,66]. mTOR plays an important role also in the maintenance of immune cell function, and the administration of rapamycin may affect immune function against infections [67]. Moreover, the long-term administration of rapamycin suppresses not only mTORC1, but also mTORC2 [68], and the attenuation of AKT activity by mTORC2 suppression may impair the functional maintenance of podocytes under disease or stress conditions [69]. Interestingly, mTORC2 inhibition rather than mTORC1 inhibition has been reported to be involved in the pathogenesis of insulin resistance and dyslipidemia by rapamycin [70,71,72]. These suggests that a more mTORC1-specific inhibitor may be a safer therapeutic agent for DKD. Furthermore, Pod-Raptor KO mice, in which mTORC1 activity is severely suppressed in podocytes, show structural abnormalities in foot processes and severe proteinuria [26]. Tubular cell-specific Raptor KO mice show impairment of endocytosis and nutrient transport [73]. These findings indicate that physiological mTORC1 activity is essential for the normal function of podocytes and tubular cells. When considering the suppression of mTORC1 as a new therapeutic approach for DKD, it is desirable to develop safe mTORC1 modulators in the future.

## 7. Conclusions

It is expected that a therapeutic approach that focuses on mTORC1 as a new therapeutic target for DKD will be promising. Rapamycin itself, an mTORC1 inhibitor, was discovered more than 40 years ago, but in recent years there has been growing interest in its pathways and new clinical applications. It is desirable to develop mTORC1 inhibitors that are more mTORC1-specific and that make it easier to regulate mTORC1 activity for clinical application as DKD therapeutics.

In summary, although there are several problems to be resolved, we believe that the correction of abnormalities in nutrient-sensing signals such as the hyperactivation of mTORC1 will become a new treatment strategy for DKD.

## Data Availability

All figures are original.
